# Supplementary material for: Female Medical Students’ Experiences of Sexism during Clinical Placements: A Qualitative Study
Source: Healthcare (Basel). 2023 Mar 31;11(7):1002. doi: 10.3390/healthcare11071002 (PMC10094657; doi:10.3390/healthcare11071002)
Supplement: Supplementary file 1 [file healthcare-11-01002-s001.zip › healthcare-2301768-supplementary.pdf]

**Supplementary materials**

Topic Guide

- ➔ Introduction
- ➔ Outline of interview format- open chat, to hear about your experiences and views, take a break at any time, don't have to answer questions, can stop at any time
- ➔ Gain verbal consent
- ➔ Confidentiality- everything discussed will remain confidential

- ➔ What year group are you currently in?
- ➔ What placements have you so far attended?
- ➔ What is your understanding of sex discrimination?
- ➔ and sexual harassment?
- ➔ Have you been witness to sexism or sexual harassment on placement?
- ➔ Have you personally experienced sexism?
- ➔ Have you personally experienced sexual harassment?
- ➔ How frequently have you experienced such encounters? (Daily, weekly, each semester, yearly)
- ➔ Who was the perpetrator? - doctor, patient, student?
- ➔ Was it in a particular specialty?
- ➔ What effect did this experience have on you? - psychological and emotional impact
- ➔ What strategies, if any, did you employ to try and cope with/manage the situation?
- ➔ How did you weigh up how to respond? (who it was/ power imbalance/ time/ concerns)
- ➔ What is your understanding of reporting procedures?
- ➔ Did you choose to report it?
- ➔ If you decided to report it, what was the outcome?
- ➔ If you didn't report it why?
- ➔ Did you experience any barriers in reporting?
- ➔ Did you experience any facilitators in reporting?
- ➔ What do you think should be done to tackle this problem?

- ➔ Summarise
- ➔ Questions or comments?
- ➔ Remind participant that they will be sent a summary of the topics arising from the transcript
- ➔ Remind about Participant Information Leaflet
- ➔ Clarify how they would like to be contacted after the interview for a check in
- ➔ Thank participant
